# Comorbidity landscape of the Danish patient population affected by chromosome abnormalities

**DOI:** 10.1038/s41436-019-0519-9

**Published:** 2019-04-25

**Authors:** Isabella Friis Jørgensen, Francesco Russo, Anders Boeck Jensen, David Westergaard, Mette Lademann, Jessica Xin Hu, Søren Brunak, Kirstine Belling

**Affiliations:** 1 Novo Nordisk Foundation Center for Protein Research, Faculty of Health and Medical Sciences, University of Copenhagen, Copenhagen, Denmark; 2 Institute for Next Generation Healthcare, Department of Genetics and Genomic Sciences, Icahn School of Medicine at Mount Sinai, New York, NY USA

**Keywords:** comorbidity, inverse comorbidity, chromosome abnormality, Down syndrome, mosaicism

## Abstract

**Purpose:**

Most chromosome abnormality patients require long-term clinical care. Awareness of mosaicism and comorbidities can potentially guide such health care. Here we present a population-wide analysis of direct and inverse comorbidities affecting patients with chromosome abnormalities.

**Methods:**

We extracted direct and inverse comorbidities for the 11 most prevalent chromosome abnormalities from the Danish National Patient Registry (covering 6.9 million patients hospitalized between 1994 and 2015): trisomy 13, 18, and 21, Klinefelter (47,XXY), triple X, XYY, Turner (45,X), Wolf–Hirschhorn, Cri-du-chat, Angelman, and Fragile X syndromes (FXS). We also performed four sub-analyses for male/female Down syndrome (DS) and FXS and non-mosaic/mosaic DS and Turner syndrome.

**Results:**

Our data cover 9,003 patients diagnosed with at least one chromosome abnormality. Each abnormality showed a unique comorbidity signature, but clustering of their profiles underlined common risk profiles for chromosome abnormalities with similar genetic backgrounds. We found that DS had a decreased risk for three inverse cancer comorbidities (lung, breast, and skin) and that male FXS and non-mosaic patients have a much more severe phenotype than female FXS and mosaic patients, respectively.

**Conclusion:**

Our study underlines the importance of considering mosaicism, sex, and the associated comorbidity profiles of chromosome abnormalities to guide long-term health care of affected patients.

## INTRODUCTION

Chromosome abnormalities result from either gain or loss of entire chromosomes (aneuploidy), or parts of chromosomes (structural abnormalities).^1^ In general, chromosome abnormalities have major, but varying, impacts. Mosaicism also exists with varying frequency.^2^ Mosaic patients may show very few clinical symptoms, yet, the impact of a mosaicism depends on the number and cell types affected.^3–5^ For aneuploidies, sex chromosome aneuploidies are more compatible with life than autosomal aneuploidies, where only three trisomies are tolerated: Patau (trisomy 13), Edwards (trisomy 18), and Down syndromes (DS) (trisomy 21). However, infants born with trisomy 13 or 18 typically die within the first weeks.^6^ Prevalent sex chromosome aneuploidies are Klinefelter syndrome (KS) (47,XXY), triple X syndrome (47,XXX), XYY syndrome (47,XYY), and Turner syndrome (TS) (45,X).

Structural abnormalities can be caused by partial chromosome deletions such as in Wolf–Hirschhorn and Cri-du-chat syndromes caused by partial deletions of the short arm of chromosome 4 and 5, respectively,^7^ or other chromosome alterations such as in Angelman syndrome (deletions or variants in *UBE3A* on chromosome 15^8^) and Fragile X syndrome (FXS) (expansion of *FMR1* on chromosome X).^9^ FXS is associated with cognitive deficits and behavioral problems, yet, females are most often compensated by the normal X chromosome and their phenotypes vary substantially.^10^

The birth rate of chromosome abnormalities is estimated to 17–31/10,000 in the European population. Many of them will need lifelong clinical care,^11^ which can be guided by knowledge of common comorbidities, defined as diseases that co-occur more frequently with a primary disease compared with controls. Previous studies have described disease course and clinical characteristics of chromosome abnormalities (reviewed by Tyler et al.^12^) including specific comorbidities. DS, KS, and TS are especially well described because numerous individuals are affected and their life expectancy is relatively high. DS is typically diagnosed at birth, TS usually shows during puberty, while KS is most often identified in adulthood when evaluating infertility. DS is the most common chromosome disorder causing intellectual disability. DS patients are often affected by comorbidities such as gastrointestinal malformations, congenital heart defects, respiratory disease, and autoimmune diseases.^13^ KS patients have a widely varying phenotype, but common characteristics are small testes, narrow shoulders, broad hips, sparse body hair, gynecomastia, and decreased verbal intelligence.^14^ It is estimated that KS causes 1–3% of all male infertility cases.^4,15,16^ Additionally, KS patients are often affected by several other comorbidities, such as osteoporosis, diabetes mellitus, cardiac diseases, and cognitive disorders.^4^ TS is the only common monosomy. The majority of TS women are infertile due to early ovarian failure, usually before puberty.^17^ Known comorbidities of TS includes cardiovascular, neurological, genitourinary, and skeletal defects.^18^

The life expectancy of aneuploidy patients is affected by their comorbidity burden. Although DS patients have a lower life expectancy than the general population, institutionalization and advantages in medical care have increased their life expectancy almost fourfold in the past 40 years.^19^ KS patients have an increased mortality mainly due to infectious, neurological, circulatory, pulmonary, and urinary tract diseases,^20^ while TS is associated with a threefold increase in overall mortality and a life expectancy reduced by up to 13 years.^21^

There is an increasing interest in studying disease–disease relationships, direct but also inverse comorbidities, defined as diseases that co-occur less frequently with a primary disease compared with controls. Knowledge of disease dependencies can provide knowledge of disease-causing or disease-protective genes and multifunctional proteins, and potentially guide disease prevention or treatment development.^22^ Central nervous system disorders have previously been associated with decreased risk of cancer.^23^ DS in particular has shown decreased risk of solid tumors, e.g. embryonal tumors, breast and skin cancer. In contrast, DS patients have increased risk of other cancers such as acute leukemia and testicular cancer.^24^ To the best of our knowledge no inverse comorbidities have been identified for the remaining ten chromosome abnormalities included in this study.

Previous studies of chromosome abnormality comorbidities have mainly focused on one patient group at a time, on direct comorbidities only, and were often based on only a few patients.^25^ Thus, any shared consequences of having an altered karyotype have yet not been studied in a data-driven manner. Here, we present a population-wide, systematic, and comparative analysis of direct and inverse comorbidities in patients with the 11 most common chromosome abnormalities in the Danish population. The results of this study can potentially guide clinicians in the long-term health care of affected patients and possibly increase our knowledge of disease genes and mechanisms.

## MATERIALS AND METHODS

### Data

Diagnoses from the Danish National Patient Registry (DNPR)^26^ covering 6.9 million patients and more than 108 million hospital encounters between 1994 and 2015 were available. The diagnoses were encoded in the International Classification of Diseases version 10 (ICD-10), which hierarchically orders diseases in 21 chapters that are subdivided into 227 blocks of similar diseases and 1,702 disease-specific level 3 codes. The level 3 codes are further subdivided with up to four additional characters to indicate etiology, anatomic site, severity, etc. Chromosome abnormalities are encoded in ICD-10 chapter XVII “Congenital malformations, deformations and chromosomal abnormalities” in the block Q90–Q99 “Chromosomal abnormalities, not elsewhere classified.” Eleven well-defined chromosome abnormalities from this block with at least 50 patients each were identified: Patau syndrome (trisomy 13), Edwards syndrome (trisomy 18), DS (trisomy 21), KS (47,XXY), triple X syndrome, XYY syndrome, TS (45,X), Wolf–Hirschhorn syndrome, Cri-du-chat syndrome, Angelman syndrome, and FXS. Four sub-analyses where also performed: female/male DS and FXS, and non-mosaic/mosaic DS and TS patients where each subgroup had at least 50 patients. Only patients with either a mosaic or a nonmosaic diagnosis in the registry were included in the subgroup analysis. See Table 1 for specific ICD-10 codes for each patient group.

**Table 1:** Number of patients, characteristics, and number of direct and inverse comorbidities for each of the 11 most prevalent chromosome abnormalities in the Danish population.

Chromosome abnormality	ICD-10 diagnosis code(s)	Number of patients	Number of direct comorbidities	Number of inverse comorbidities	Median age at first diagnosis (SD)	Median age at death (SD)	Median follow-up years (SD)
Patau syndrome (trisomy 13)	Q91.4, Q91.5, Q91.6, Q91.7	72	21	0	0.00 (12.47)	0.02 (12.93) (*n* = 55)	0.08 (7.38)
Edwards syndrome (trisomy 18)	Q91.0, Q91.1, Q91.2, Q91.3	175	29	0	0.00 (14.72)	0.02 (9.64) (*n* = 126)	0.18 (7.58)
Down syndrome (trisomy 21)	Q90	3,474	236	56	21.29 (23.00)	56.65 (19.66) (*n* = 931)	16.20 (6.64)
Female Down syndrome		1,601	181	34	22.36 (23.23)	56.37 (19.23) (*n* = 438)	15.87 (6.63)
Male Down syndrome		1,873	194	32	20.30 (22.79)	57.18 (20.06) (*n* = 493)	16.41 (6.65)
Non-mosaic Down syndrome	Q90.0	1,050	211	10	0.24 (17.69)	51.31 (25.56) (*n* = 166)	16.90 (6.27)
Mosaic Down syndrome	Q90.1	62	15	0	7.89 (19.73)	56.65 (23.78) (*n* = 11)	18.25 (6.13)
Klinefelter syndrome (47,XXY)	Q98.0, Q98.1, Q98.2, Q98.4	764	88	1	28.12 (18.11)	58.32 (15.89) (*n* = 53)	18.97 (5.34)
Triple X syndrome	Q97.0	65	6	0	3.13 (11.74)	NA^a^	13.35 (6.62)
XYY syndrome	Q98.5	81	12	0	12.50 (12.75)	NA^a^	17.95 (5.04)
Turner syndrome (45,X)	Q96	870	119	9	16.75 (19.19)	61.41 (20.89) (*n* = 86)	18.86 (5.67)
Non-mosaic Turner syndrome	Q96.0	241	48	0	12.31 (15.08)	55.14 (22.64) (*n* = 14)	19.70 (5.30)
Mosaic Turner syndrome	Q96.3	58	1	0	10.36 (14.70)	NA^a^	12.49 (6.28)
Wolf–Hirschhorn syndrome	Q93.3	51	37	0	2.02 (12.06)	0.62 (15.31) (*n* = 8)	10.79 (7.15)
Cri-du-chat syndrome	Q93.4	62	32	0	6.28 (18.24)	24.29 (22.58) (*n* = 9)	15.69 (7.08)
Angelman syndrome	Q93.5C	80	33	0	5.09 (14.95)	NA^a^	13.88 (5.93)
Fragile X syndrome	Q99.2	206	29	0	7.64 (19.27)	68.88 (22.44) (*n* = 7)	16.77 (6.13)
Female Fragile X syndrome		58	3	0	10.47 (19.35)	NA^a^	14.54 (6.17)
Male Fragile X syndrome		148	22	0	6.61 (19.29)	65.04 (25.22) (*n* = 5)	17.27 (6.10)

*ICD-10* International Classification of Diseases version 10.

^a^Fewer than five patients were available.

### Extraction of direct and inverse comorbidities

Direct and inverse comorbidities were identified as the over- or under-occurrence of a disease in chromosome abnormality patients compared with sex and year of a birth-matched control group. A control population was randomly drawn for each patient group from a pool of patients not having any diagnoses in block Q90–Q99 “Chromosomal abnormalities, not elsewhere classified.” Every patient was matched to 20 controls to have enough controls to pick up rare diseases, but still not give the test arbitrarily strong statistical power.^25^

Association strength was assessed using the relative risk (RR) measure. A pseudo count of 1 was added to the calculation to avoid RR overestimation of disease associations for rare diseases.^25^ Thus, let *A*_*t*_ be the number of chromosome abnormality patients and *A*_*X*_ the number of these patients also diagnosed with disease X. Likewise, let *C*_*t*_ be the number of control patients and *C*_*X*_ the number of control patients also diagnosed with disease X. Then RR is defined as:

$${\mathrm{Relative}}\,{\mathrm{Risk}}\left( {{\mathrm{RR}}} \right) = \frac{{\left( {{\mathrm{A}}_{\mathrm{X}} + 1} \right)/\left( {{\mathrm{A}}_{\mathrm{t}} + 1} \right)}}{{\left( {{\mathrm{C}}_{\mathrm{X}} + 1} \right)/\left( {{\mathrm{C}}_{\mathrm{t}} + 1} \right)}}$$

A two-sided Fisher’s exact test was applied to calculate *p* values, which were corrected for multiple testing using the Benjamini–Hochberg procedure. For each direct and inverse comorbidity, we required a minimum of five patients diagnosed with disease X, a RR ≥ 1.5 or RR ≤ 0.5, and false discovery rate (FDR) ≤0.05.^25^ Hierarchical clustering with Euclidean distance, using R3.3.1 and the ComplexHeatmap package, of the RRs for all direct and inverse comorbidity profiles was used to find common patterns.

The larger a population is, the more disease co-occurrences are tested, and thus more comorbidities are potentially identified. To account for the large difference in patient number between non-mosaic and mosaic DS and TS patients (Table 1), we randomly split the non-mosaic patients into groups of a similar size to the mosaic patient group and following identified direct and inverse comorbidities for the two groups now of similar size.

### Survival analysis

A Cox proportional hazards regression model was applied to compare mortality of DS patients with 20 sex- and year of birth–matched controls. A multivariate Cox analysis was used to model survival time as a function of sex and group using survival and survminer in R3.3.1. Survival difference between DS patients and controls was visualized using Kaplan–Meier curves. The proportional hazards assumption in the Cox regression model was tested using scaled Schoenfeld residuals. Additionally, a two-sided chi-square test was used to assess the difference in incidence rate of three malignant solid cancers between DS patients and sex- and age-matched controls.

### Change in comorbidity burden and life expectancy

Disease distributions across the ICD-10 chapters were calculated for DS, KS, and TS. The percentage of the patient group affected with diseases was summarized for each chapter and visualized according to the age at first diagnosis. The relative change in occurrence of comorbidities over time was calculated for the DS, KS, and TS comorbidities for which at least 10% of the patients had the diagnosis. The prevalence of each comorbidity was calculated for each year in the registry and adjusted for the number of DS, KS, or TS patients in the specific year, respectively. Because the prevalence does not take the potential age change of each patient group into account it should be considered relative. A loess (locally weighted smoothing) regression was used to fit a smooth curve to discover changes in comorbidity prevalence over time.

Life expectancy was calculated as the median age of death for each patient group each year. Linear regression was fitted to model the overall change in life expectancy. Additionally, the number of births of DS, KS, and TS patients and the median age of the patients in the registry were calculated year-wise. The loess regression and linear regression were done using the R packages ggplot2 and stats (R version 3.3.1).

### Data and materials approval

This study was approved by the Danish Data Protection Agency, Copenhagen (ref: SUND-2016-83) and Statens Serum Institut (ref: FSEID-00003092).

## RESULTS

### Direct and inverse comorbidities of chromosome abnormality patients

In total, 9,003 patients in DNPR were diagnosed with at least one chromosome abnormality in the period 1994–2015. Direct (50% increased risk, RR ≥ 1.5) and inverse (50% decreased risk, RR ≤ 0.5) comorbidities were systematically identified for the 11 most prevalent chromosome abnormalities by comparison of disease frequency in a sex- and year of birth–matched background population. In total, 338 unique direct and 60 unique inverse comorbidities were identified for the 11 chromosome abnormalities. Direct comorbidities were identified for all 11 chromosome abnormalities. Yet, significant inverse comorbidities were only identified for DS, KS, and TS. Tables S1 and S2 contain RR, FDR, and median age at first diagnosis for all analyses.

Direct and inverse comorbidities were also identified for the four subgroups: female/male DS and FXS, separately, and non-mosaic/mosaic DS and TS, separately. Both significant direct and inverse comorbidities were identified for male and female DS (Tables S3 and S4). Only direct comorbidities were identified for male and female FXS, and only three comorbidities for female FSX (Table S5). Only patients with specified karyotype were included in the comorbidity analyses for non-mosaic and mosaic patients. In the comorbidity analyses for the general patient population also patients with an unspecified karyotype were included. Direct and inverse comorbidities were identified for non-mosaic DS patients, whereas only direct comorbidities were identified for mosaic DS patients (Table S6). Only direct comorbidities were identified for non-mosaic and mosaic TS patients, and only one direct comorbidity was identified for mosaic TS patients (Table S7). As for the comorbidity analyses of the full patient populations we also included median age at first diagnosis for all the subgroup analyses, which can be of interest for comparison between female/male and non-mosaic and mosaic patients. The specific ICD-10 codes for all patient groups and the median age of chromosome abnormality diagnosis are stated in Table 1. All supplementary tables with inverse and direct comorbidities are available for download (Tables S1–S7) or in an online, searchable form here: http://chrom-abn.cpr.ku.dk/.

**Fig. 1:**
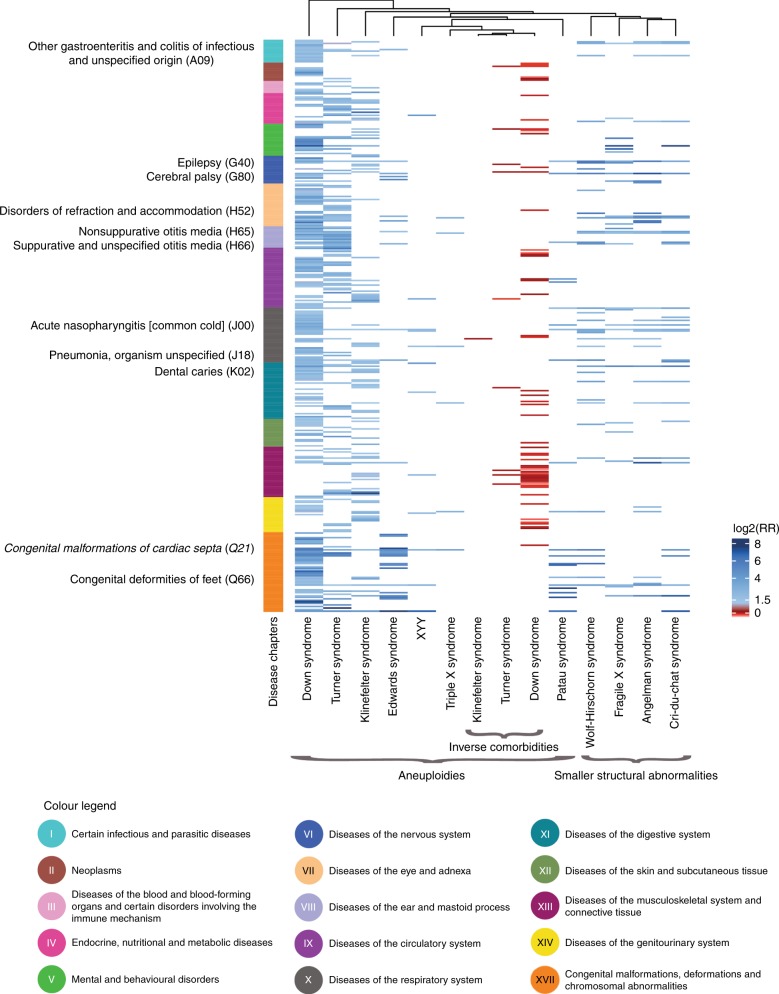
**Comorbidity signatures for the 11 most prevalent chromosome abnormalities in the Danish population.** The direct (*n* = 11) and inverse (*n* = 3) comorbidity profiles are grouped vertically according to International Classification of Diseases version 10 (ICD-10) chapter (color scale explained at the bottom) and hierarchically clustered horizontally based on their relative risk (RR) signatures (color scale right). To increase the quality of the clustering and visualization, log2(RR +1) was used. Each chromosome abnormality had a specific signature, but they clustered in two distinct groups: aneuploidies (left) and smaller structural abnormalities (right). The one direct comorbidity in common between all aneuploidies is written in italics to the left of the disease chapter bar and the ten direct comorbidities in common between the four smaller structural abnormalities are listed as well. All direct and inverse comorbidities are available in Tables S1 and S2, respectively.

All chromosome abnormalities had a unique comorbidity signature and none of the direct or inverse comorbidities were shared between all 11 patient groups. Yet, most chromosome abnormality patients had high RR of diseases from chapter XVII “Congenital malformations, deformations and chromosomal abnormalities” and chapter V “Mental and behavioral disorders.” Aneuploidies only had one direct comorbidity in common, “Congenital malformations of cardiac septa” (Q21), whereas the four smaller structural abnormalities had ten shared direct comorbidities. Inverse comorbidities were calculated for all 11 chromosome abnormalities, but were only identified for DS (*n* = 56), KS (*n* = 1), and TS (*n* = 9). Clustering the 14 RR profiles left the aneuploidies in one group and smaller structural abnormalities in another (Fig. 1), which confirms common risk profiles for chromosome abnormalities with similar genetic backgrounds.

**Fig. 2:**
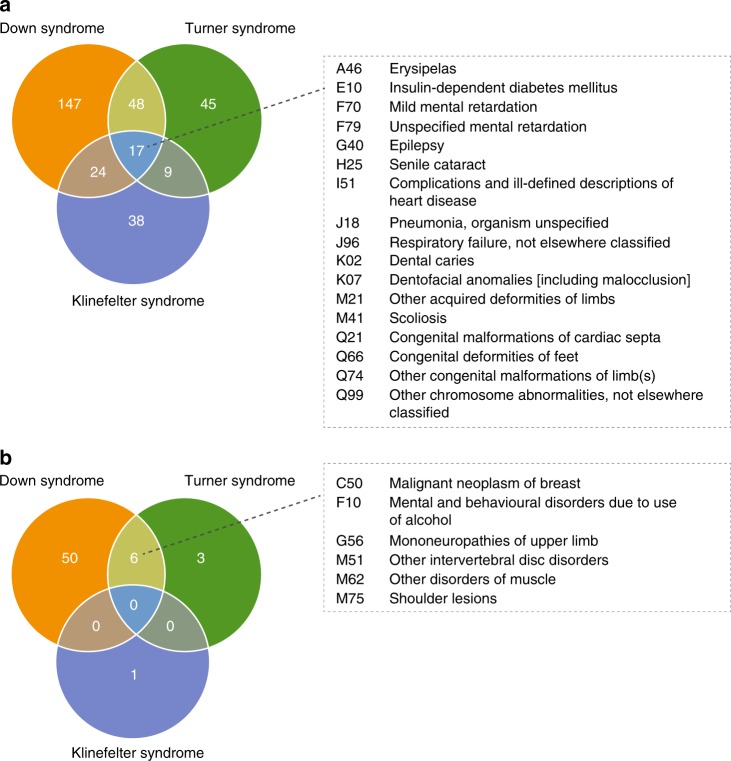
**Venn diagrams with the number of shared direct and inverse comorbidities between Down syndrome (DS), Klinefelter syndrome (KS), and Turner syndrome (TS) patients, respectively.** **a** A total of 17 direct comorbidities were shared between DS, KS, and TS. **b** No inverse comorbidities were shared between DS, KS, and TS, but six inverse comorbidities were shared between DS and TS. The shared comorbidities are listed by International Classification of Diseases version 10 (ICD-10) code and named in the boxes to the right.

Most chromosome abnormality patients were diagnosed with DS, KS, or TS and, as expected, most comorbidities were identified for these. In total, 17 direct comorbidities were shared between DS, KS, and TS, and numerous comorbidities were shared pairwise between the three (Fig. 2a). No inverse comorbidity was shared between DS, KS, and TS, only six inverse comorbidities overlapped between DS and TS (Fig. 2b). In total, 56 inverse comorbidities were identified for DS, only “Chronic disease of tonsils and adenoids” (J35) (RR = 0.46) was observed as inverse comorbidity for KS, and nine inverse comorbidities were identified for TS (Table 1 and Table S2). Please see Text S1 for an elaborate presentation on the shared direct and inverse comorbidities of DS, KS, and TS.

### Cancer risk for Down syndrome patients

We discovered six direct comorbidities of DS from chapter II “Neoplasms” (including one benign neoplasm): “Other leukemias of specified cell type” (C94) (RR = 24.41), “Leukemia of unspecified cell type” (C95) (RR = 12.07), “Myeloid leukemia” (C92) (RR = 7.63), “Lymphoid leukemia” (C91) (RR = 4.46), “Malignant neoplasm of testis” (C62) (RR = 2.77), and “Benign neoplasm of mouth and pharynx” (D10) (RR = 2.09). Contrarily, we observed six inverse comorbidities related to cancer including three benign and three malignant cancer types: “Malignant neoplasm of bronchus and lung” (C34) (RR = 0.25), “Malignant neoplasm of breast” (C50) (RR = 0.26), and “Other malignant neoplasms of skin” (C44) (RR = 0.25) (see Table S2), which supports earlier hypotheses.^24^ These findings could be due to the shorter life expectancy of DS patients confirmed by a multivariate Cox model (Table S8). Our data showed that more than 80% of DS patients were alive at age 50, whereas very few survived until age 75 (Fig. S1). To take this shorter life expectancy into account, all DS patients were matched to 20 random controls with the same age and sex where the incidence rate of the three malignant cancers were compared with a chi-squared test (Table S9). Still, all three cancers appeared significantly less in DS patients, supporting the hypothesis of a solid cancer protection phenotype of DS.

### Variance in diagnosis age between aneuploidies

**Fig. 3:**
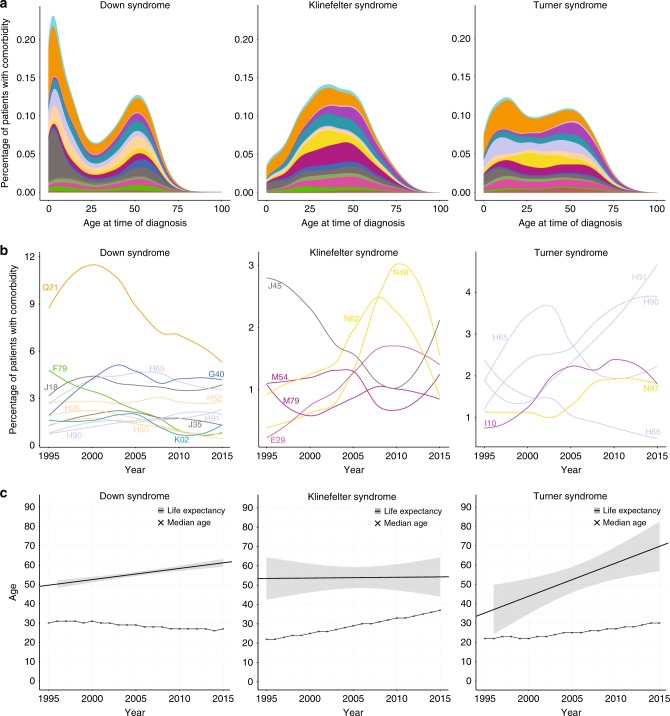
**Comorbidity distributions and life expectancy of Down syndrome (DS), Klinefelter syndrome (KS), and Turner syndrome (TS) patients.** **a** The age at first diagnosis (*x-*axis) versus percentage of patients diagnosed (*y*-axis) for DS, KS, and TS. DS patients are usually diagnosed in early life, TS patients are usually diagnosed during childhood, and KS patients are usually diagnosed at the age of trying to conceive, with the peak around age 30. See Fig. 1 for color scheme. **b** The relative comorbidity burden over the past 20 years for DS, KS, and TS. Again, the colors correspond to the International Classification of Diseases version 10 (ICD-10) chapters (see Fig. 1). The names of the comorbidities displayed are as follows: E29 = Testicular dysfunction; F79 = Unspecified mental retardation; G40 = Epilepsy; H26 = Other cataract; H50 = Other strabismus; H52 = Disorders of refraction and accommodation; H65 = Nonsuppurative otitis media; H66 = Suppurative and unspecified otitis media; H90 = Conductive and sensorineural hearing loss; H91 = Other hearing loss; I10 = Essential (primary) hypertension; J18 = Pneumonia, organism unspecified; J35 = Chronic diseases of tonsils and adenoids; J45 = Asthma; K02 = Dental caries; M54 = Dorsalgia; M79 = Other soft tissue disorders, not elsewhere classified; N46 = Male infertility; N62 = Hypertrophy of breast; N97 = Female infertility; and Q21 = Congenital malformations of cardiac septa. **c** The change in life expectancy and median age of the patients in the registry per year for DS, KS, and TS. The life expectancy for DS and TS has increased over the past 20 years whereas the life expectancy for KS has not changed. The median age of the patients in the registry has slightly decreased for DS, but increased for KS and TS.

To better understand the unique comorbidity signatures of the three most prevalent aneuploidies, we studied the age at comorbidity diagnosis for each of the three patient groups (Fig. 3a). DS, KS, and TS had comorbidities from many of the same ICD-10 chapters, but the age at diagnosis differed: DS was typically diagnosed at birth, which was seen from the peak in chapter XVII “Congenital malformations, deformations and chromosomal abnormalities” (orange) in very early life. The age at KS diagnosis varied but peaked around the age of 30 with several diseases from chapter XIV “Diseases of the genitourinary system” (yellow) including “Male infertility” (N46). TS patients were usually diagnosed during childhood and often had comorbidities from chapter VIII “Diseases of the ear and mastoid process” (purple). Additionally, in early adulthood several TS patients were diagnosed with chapter XIV “Diseases of the genitourinary system” (yellow) including “Female infertility” (N97).

### Evolution of comorbidity burden and life expectancy

The comorbidity burden has changed over the past 20 years for all three common aneuploidies. Figure 3b shows the comorbidities affecting at least 10% of the DS, KS, and TS patients, respectively. The incidence rate of “Congenital malformations of cardiac septa” (Q21) has decreased for DS. For KS, the relative incidence of “Male infertility” (N46), “Testicular dysfunction” (E29), and “Hypertrophy of breast” (N62) have increased, but then dropped again recently, while the occurrence of “Asthma” (J45) has fluctuated during this period. For TS patients the occurrence of hearing loss (H90 and H91) has increased, while the incidence of otitis media (H65 and H66) has decreased. Still, the percentage of the patient population affected by these comorbidities is relatively low, especially for KS and TS.

The life expectancy of DS and TS has increased over the past 20 years by 9 and 35 years, respectively, whereas the life expectancy of KS has been stable (Fig. 3c). The median age of the DS patients in the registry has slightly decreased, whereas it has increased for KS and TS with 15 and 8 years, respectively. The birth rate for DS has also dramatically decreased during the past 20 years from around 70 patients a year to around 35. The drop of DS births from 2004 to 2005 reflects new Danish guidelines offering prenatal screening including a risk assessment for DS to all pregnant women.^27^ The birth rate has only slightly decreased for KS and TS (Fig. S2).

### Sex influences incidence of comorbidities

**Fig. 4:**
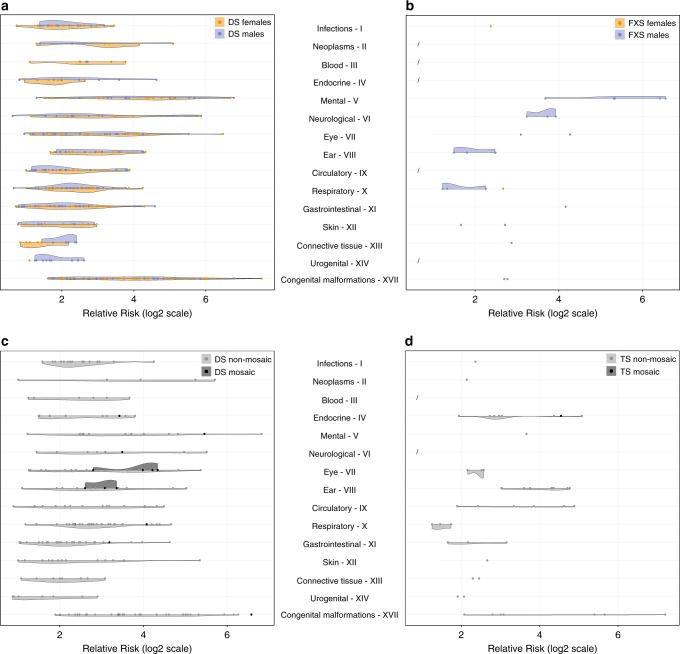
**Comorbidities per International Classification of Diseases version 10 (ICD-10) chapter for the four subgroup comparisons: female and male Down syndrome (DS) and fragile X syndrome (FXS) and non-mosaic and mosaic DS and Turner syndrome (TS).** Significant comorbidities were extracted from each subgroup and summarized by disease chapter. Each dot represents a comorbidity (orange for females, blue for males, gray for non-mosaic, and black for mosaic) with a certain relative risk (RR). **a** Female and male DS were both associated with several comorbidities, but female DS had more comorbidities from chapter III “Diseases from the blood and blood-forming organs and certain disorders involving the immune mechanism,” while male DS had more diseases from chapter XIV “Diseases of the genitourinary system.” **b** Only three significant comorbidities were observed for female FXS, while males were more severely affected, especially with diseases from chapter V “Mental and behavioral disorders” and chapter VI “Diseases of the nervous system.” (**c**,**d**) Both DS and TS non-mosaic patients show a more severe phenotype with 211 and 49 significant comorbidities, respectively, compared with DS and TS mosaic patients, with only 16 and 1 comorbidities, respectively. Titles of disease chapters are shortened (see Fig. 1). See Tables S3–S7 for more information.

Enough DS and FXS patients (minimum 50 patients) in the registry allowed us to perform analyses of direct and inverse comorbidities for female and male patients separately. In total, 181 and 194 direct comorbidities were found for female and male DS, respectively (Fig. 4a and Table S3). In fact, female and male DS patients share more direct comorbidities (*n* = 145) than they have specific ones (female *n* = 36 and male *n* = 49) (Fig. S3a). Examples of female DS-specific direct comorbidities were “Lymphoid leukemia” (C91), “Benign neoplasm of mouth and pharynx” (D10), and “Edwards' syndrome and Patau’s syndrome” (Q91), and examples of male DS-specific direct comorbidities were “Asthma” (J45) and “Cleft palate” (Q35). Further, 34 and 32 inverse comorbidities were identified for female and male DS, respectively, of which 19 were shared (Table S4). Female DS-specific inverse comorbidities included “Depressive episode” (F32) and “Irritable bowel syndrome” (K58) and male DS-specific inverse comorbidities included “Chronic ischemic heart disease” (I25) and “Acute myocardial infarction” (I21).

The phenotypic impact of FXS varies between males and females.^10^ Our analysis confirmed that FXS males suffer from more comorbidities (*n* = 22) than FXS females (*n* = 3) (Fig. 4b and Table S5). The only common direct comorbidity was “Nonsuppurative otitis media” (H65) (Fig. S3b), whereas male FXS-specific direct comorbidities included “Mental retardation” (F70, F71, F79) and “Epilepsy” (G40).

### Non-mosaic patients have a more severe phenotype compared with mosaic patients

For DS and TS, high enough patient counts and a clear ICD-10 classification allowed us to perform comorbidity analyses on non-mosaic/mosaic patients separately. A total of 211 and 15 direct comorbidities were identified for non-mosaic and mosaic DS patients, respectively (Fig. 4c and Table S6). The mosaic DS patients did not have any unique direct comorbidities (Fig. S3c). Ten inverse comorbidities were identified for non-mosaic DS patients including “Essential (primary) hypertension” (I10) and “Angina pectoris” (I20). No inverse comorbidities were identified for mosaic DS patients.

A total of 48 and 1 direct comorbidities were identified for non-mosaic and mosaic TS patients, respectively (Fig. 4d and Table S7). The one direct comorbidity for mosaic TS patients, “Other endocrine disorders” (E34) was shared between non-mosaic and mosaic patients (Fig. S3d). For both DS and TS, the number of non-mosaic patients highly exceeded the number of mosaic patients (Table 1). To check whether this skewed patient numbers and influenced the number of identified comorbidities, we subdivided the non-mosaic patients into groups of similar size to the mosaic patients available and identified comorbidities for these groups (Table S10). It was apparent from these analyses that the number of patients affects the number of identified comorbidities. Still, non-mosaic patients had a much more severe phenotype than mosaic patients. This was also reflected by the earlier age at diagnosis for non-mosaic compared with mosaic patients (Fig. S4).

## DISCUSSION

We present a population-wide and comparative analysis on direct and inverse comorbidities of the 11 most prevalent chromosome abnormalities in the DNPR that contains patients of high ancestral homogeneity. This registry contains 6.9 million patients giving strong statistical power to perform epidemiological studies. Each abnormality showed a unique comorbidity signature, but clustering of the 14 RR profiles (11 direct and 3 inverse) underlined common risk profiles for chromosome abnormalities with similar genetic backgrounds.

Most chromosome abnormality patients in the registry suffered from DS, KS, or TS, and, thus, most comorbidities were identified for these three. DS, KS, and TS patients all had an increased RR of “Insulin-dependent diabetes mellitus” (E10) with TS patients having the highest RR of 4.3. KS and TS patients also had an increased risk of “Non–insulin-dependent diabetes mellitus” (E11) and “Unspecified diabetes mellitus” (E14) (see Table S1 for RRs and *p* values). This result was consistent with the fact that type 1 diabetes is more prevalent in DS patients compared with the general population,^28^ while few cases of type 2 diabetes have been reported.^29^ However, an increased risk for KS and TS of type 2 diabetes has previously been reported, compared with very few type 1 diabetes cases.^30,31^

Our results indicate that DS patients have lower risk of some solid tumors (lung, breast, and skin), referred to as inverse cancer comorbidities, which supports previous findings.^32^ However, this is a controversial topic^33^ and the molecular mechanism of the protective effect against solid tumors is still poorly understood. Extensive epidemiological studies point out inverse cancer comorbidities in patients affected by central nervous system (CNS) disorders and several other diseases such as DS, Parkinson disease, schizophrenia, Alzheimer disease, multiple sclerosis, and Huntington disease.^33^ It has been reported that DS is among the CNS disorders most strongly associated with cancer, in particular acute leukemia, testicular cancer, and gastrointestinal tumors.^24^

The comorbidity burden and life expectancy are interconnected. The comorbidity burden can increase due to patients living longer and, vice versa, the life expectancy can go up due to better diagnosis and treatment of certain comorbidities. All three common aneuploidies showed a change in the relative comorbidity burden over the past 20 years (Fig. 3b). The change in the relative comorbidity burden can be explained by two hypotheses: (1) the actual prevalence of comorbidity burden changes, or (2) the health system is improving at monitoring, discovering, and preventing the disease.

We performed comorbidity analyses for four subgroups of patients: female/male DS and FXS and non-mosaic/mosaic DS and TS. Female FXS only had three direct comorbidities while males showed a more severe phenotype characterized by 22 comorbidities. This result can be justified by the presence of an extra X chromosome without *FMR1* expansion for females.^10^ Because the *FMR1* gene is essential for cognitive development, it has been shown that FXS patients commonly experience several mental disorders.^34^ This evidence is consistent with our results of enrichment of direct comorbidities in males such as “Mild mental retardation” (F70), “Moderate mental retardation” (F71), “Unspecified mental retardation” (F79), and “Pervasive developmental disorders” (F84). Further, we observed the commonly known, more severe phenotype of non-mosaic compared with mosaic patients.

DS patients often show an overexpression of amyloid precursor protein (APP), which is located on chromosome 21. APP generates β-amyloid, which is the primary component of the amyloid plaques found in DS and Alzheimer disease (AD) brains.^35^ It has been shown that DS patients by the age of 40 have neuropathological changes that are consistent with AD,^36^ thus DS patients tend to develop dementia 10–20 years earlier than the general population.^35^ This was also evident from our analysis where non-mosaic patients have a RR of 31.39 of developing AD and the median age of onset was 53 years (Table S6). AD was not a comorbidity of mosaic DS patients.

Most of the patients in DNPR were diagnosed with the unspecified diagnosis, thus the subtype of the chromosome abnormality (non-mosaic/mosaic) was not specified. Our results underline the importance of taking the genotype into consideration upon consultation of chromosome abnormality patients. Even though chromosome abnormalities cannot currently be cured, medical guidance and treatment can make it easier to handle these syndromes and relieve some of the symptoms and comorbidities. Knowledge of the genotype and the comorbidity risk profile is important to help guide clinicians when diagnosing, subgrouping, and treating these patients. Furthermore, the differences and similarities in comorbidity profiles of chromosome abnormality patients might assist in uncovering disease mechanisms to understand the different comorbidity profiles.

## Supplementary information

Figure S1

Figure S2

Figure S3

Figure S4

Table S1

Table S2

Table S3

Table S4

Table S5

Table S6

Table S7

Table S8

Table S9

Table S10

Supplementary text
