# Supplementary material for: Comorbidity landscape of the Danish patient population affected by chromosome abnormalities
Source: Genet Med. 2019 Apr 25;21(11):2485–95. doi: 10.1038/s41436-019-0519-9 (PMC6831512; doi:10.1038/s41436-019-0519-9)

Comorbidity landscape of the Danish patient population affected by chromosome abnormalities

Isabella Friis Jørgensen, MSc[1, #], Francesco Russo, PhD[1, #], Anders Boeck Jensen, PhD[2], David Westergaard, PhD[1], Mette Lademann, PhD[1], Jessica Xin Hu, PhD[1], Søren Brunak, PhD[1], Kirstine Belling, PhD[1, *]

Figure S1. Mortality of DS patients and matched controls. Kaplan-Meier curves showed a significantly lower life expectancy for DS patients (orange) compared to a sex and year of birth matched control group (green).

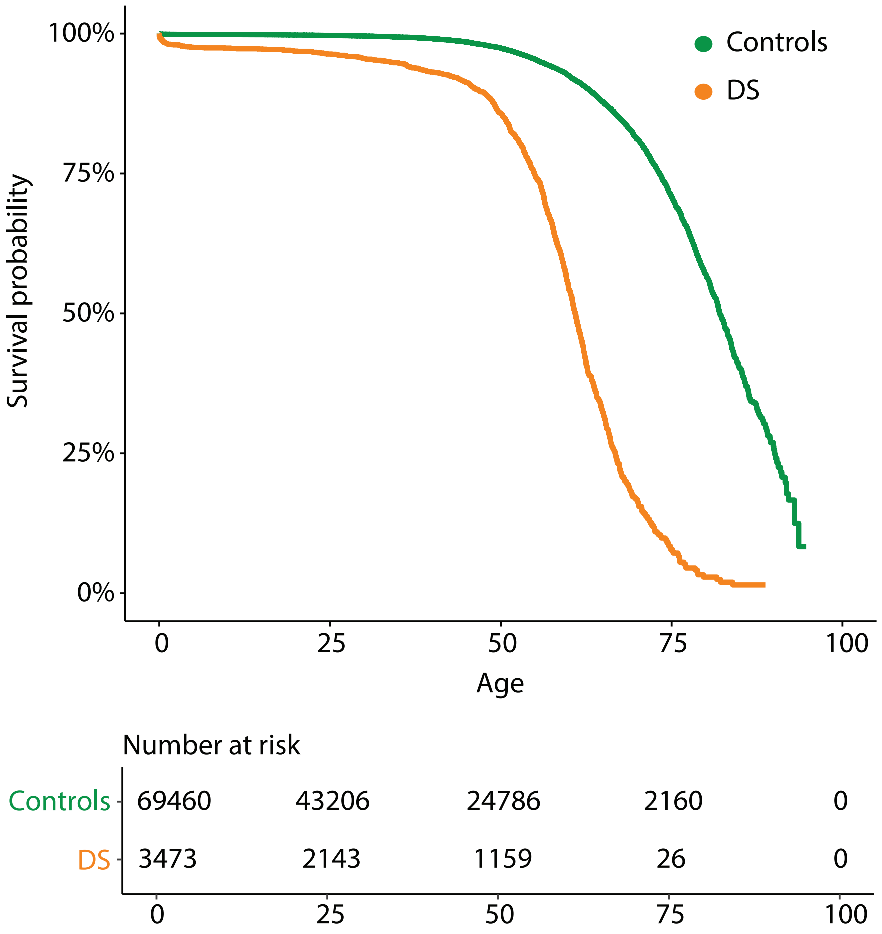

Supplement: Supplementary file 1 — Figure S1 [file 41436_2019_519_MOESM1_ESM.pdf]
