# Supplementary material for: Comorbidity landscape of the Danish patient population affected by chromosome abnormalities
Source: Genet Med. 2019 Apr 25;21(11):2485–95. doi: 10.1038/s41436-019-0519-9 (PMC6831512; doi:10.1038/s41436-019-0519-9)

Comorbidity landscape of the Danish patient population affected by chromosome abnormalities

Isabella Friis Jørgensen, MSc[1, #], Francesco Russo, PhD[1, #], Anders Boeck Jensen, PhD[2], David Westergaard, PhD[1], Mette Lademann, PhD[1], Jessica Xin Hu, PhD[1], Søren Brunak, PhD[1], Kirstine Belling, PhD[1, *]

Figure S2. Birth rate for Down syndrome (DS), Klinefelter syndrome (KS) and Turner syndrome (TS) patients the past 20 years. The birth rate for DS (orange) has dramatically decreased during the past 20 years from around 70 patients a year to around 35. The birth rate has only slightly decreased for KS (blue) and TS (green). KS and TS are typically diagnosed in puberty and adulthood, thus many of the patients being born the past 20 years, might not be diagnosed yet. We required a minimum of five patients each year to include the actual number of new-borns.

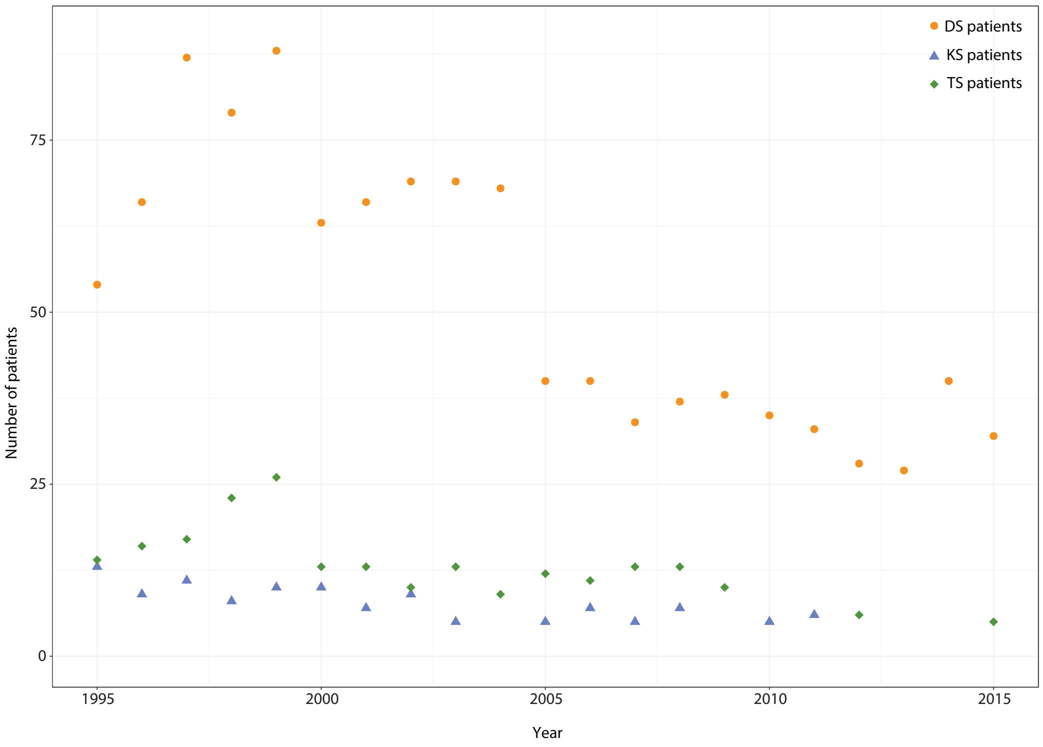

Supplement: Supplementary file 2 — Figure S2 [file 41436_2019_519_MOESM2_ESM.pdf]
