# Supplementary material for: Comorbidity landscape of the Danish patient population affected by chromosome abnormalities
Source: Genet Med. 2019 Apr 25;21(11):2485–95. doi: 10.1038/s41436-019-0519-9 (PMC6831512; doi:10.1038/s41436-019-0519-9)

Comorbidity landscape of the Danish patient population affected by chromosome abnormalities

Isabella Friis Jørgensen, MSc[1, #], Francesco Russo, PhD[1, #], Anders Boeck Jensen, PhD[2], David Westergaard, PhD[1], Mette Lademann, PhD[1], Jessica Xin Hu, PhD[1], Søren Brunak, PhD[1], Kirstine Belling, PhD[1, *]

Figure S3. Venn diagrams of the four sub-group analysis of (A) male/female Down syndrome (DS), (B) male/female Fragile X syndrome (FXS), (C) non-mosaic/mosaic DS, and (D) non-mosaic/mosaic Turner syndrome (TS).

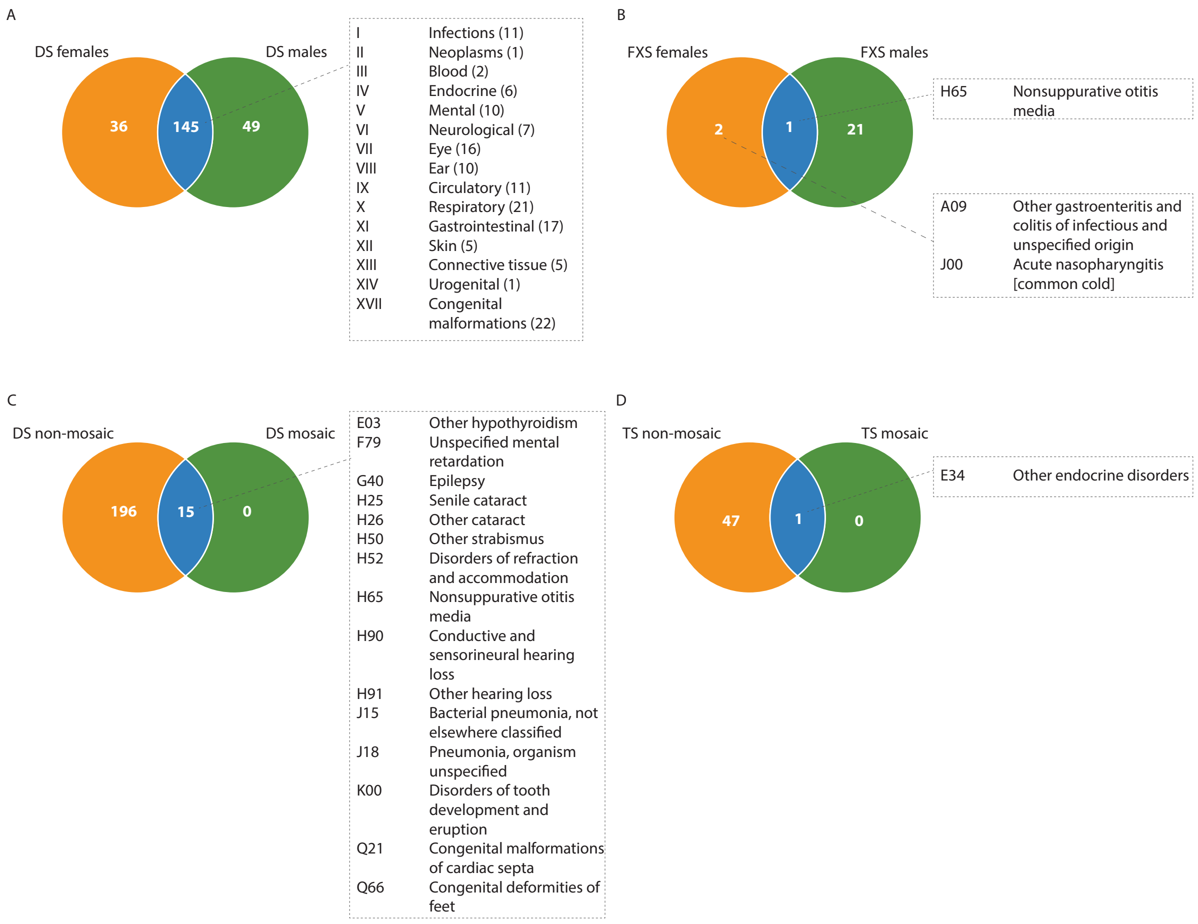

Supplement: Supplementary file 3 — Figure S3 [file 41436_2019_519_MOESM3_ESM.pdf]
