# Supplementary material for: Comorbidity landscape of the Danish patient population affected by chromosome abnormalities
Source: Genet Med. 2019 Apr 25;21(11):2485–95. doi: 10.1038/s41436-019-0519-9 (PMC6831512; doi:10.1038/s41436-019-0519-9)

Comorbidity landscape of the Danish patient population affected by chromosome abnormalities

Isabella Friis Jørgensen, MSc[1, #], Francesco Russo, PhD[1, #], Anders Boeck Jensen, PhD[2], David Westergaard, PhD[1], Mette Lademann, PhD[1], Jessica Xin Hu, PhD[1], Søren Brunak, PhD[1], Kirstine Belling, PhD[1, *]

Figure S4. Distribution of age at diagnosis of non-mosaic and mosaic Down syndrome (DS) and Turner syndrome (TS) patients. (A) Density distribution of age at first DS diagnosis for non-mosaic and mosaic patients. (B) Density distribution of age at first TS diagnosis for non-mosaic and mosaic patients.

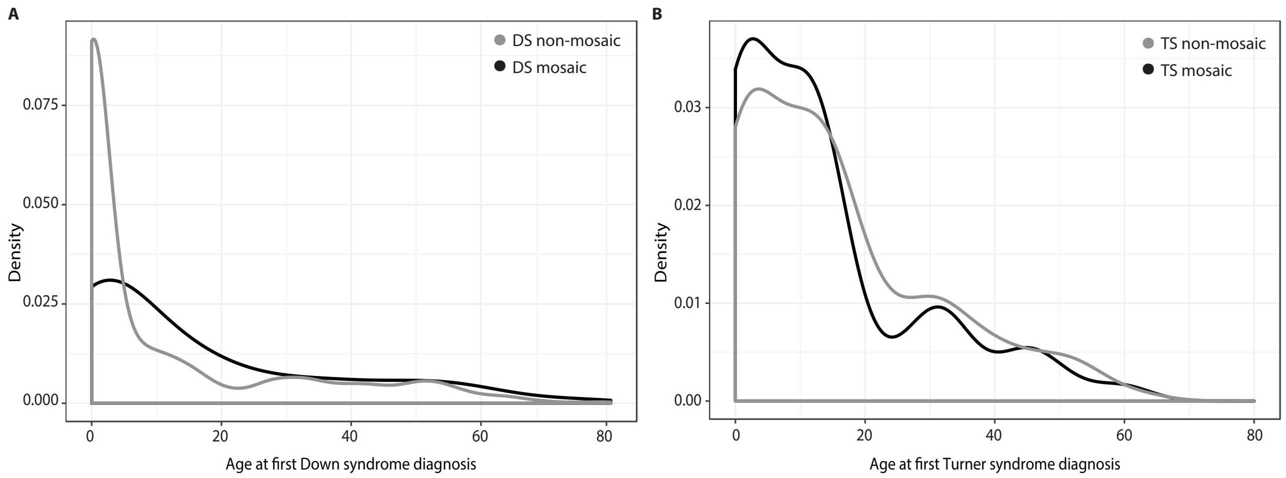

Supplement: Supplementary file 4 — Figure S4 [file 41436_2019_519_MOESM4_ESM.pdf]
