# Supplementary material for: Comorbidity landscape of the Danish patient population affected by chromosome abnormalities
Source: Genet Med. 2019 Apr 25;21(11):2485–95. doi: 10.1038/s41436-019-0519-9 (PMC6831512; doi:10.1038/s41436-019-0519-9)
Supplement: Supplementary file 12 — Table S8 [file 41436_2019_519_MOESM12_ESM.pdf]

Supplementary information

Comorbidity landscape of the Danish patient population affected by chromosome abnormalities

---

Isabella Friis Jørgensen, MSc[1, #], Francesco Russo, PhD[1, #], Anders Boeck Jensen, PhD[2], David Westergaard, PhD[1], Mette Lademann, PhD[1], Jessica Xin Hu, PhD[1], Søren Brunak, PhD[1], Kirstine Belling, PhD[1, *]

Table S8. Multivariate Cox analysis comparing life expectancy of Down syndrome (DS) patients and matched controls. A multivariate Cox proportional hazard regression model was used to model survival time as a function of sex and whether individuals were DS patients or controls. The proportional hazards assumption in the Cox regression model showed no significant deviations in any of the covariates**.** Beta coefficients, the effect sizes (given as hazard ratios) and statistical significance for each of the variables and their joint impact on overall survival are seen in the table. All variables have positive beta coefficients, thus being male and being DS patient is associated with poorer survival. HR = Hazard Ratio; CI = Confidence Interval.

	**Beta Coefficient**	**HR (95% CI)**	**Wald test**	**P-value**
Sex (male)	0.37	1.45 (1.37-1.54)	161.6	$>2.2e^{-16}$
Group (DS)	2.04	7.68 (7.14-8.26)	3016	$>2.2e^{-16}$
