# Supplementary material for: Comorbidity landscape of the Danish patient population affected by chromosome abnormalities
Source: Genet Med. 2019 Apr 25;21(11):2485–95. doi: 10.1038/s41436-019-0519-9 (PMC6831512; doi:10.1038/s41436-019-0519-9)
Supplement: Supplementary file 13 — Table S9 [file 41436_2019_519_MOESM13_ESM.pdf]

Supplementary information

Comorbidity landscape of the Danish patient population affected by chromosome abnormalities

---

Isabella Friis Jørgensen, MSc[1, #], Francesco Russo, PhD[1, #], Anders Boeck Jensen, PhD[2], David Westergaard, PhD[1], Mette Lademann, PhD[1], Jessica Xin Hu, PhD[1], Søren Brunak, PhD[1], Kirstine Belling, PhD[1, *]

Table S9. Two-sided chi-square test comparing incidence of three solid cancers in Down syndrome (DS) patients and matched controls. To account for shorter life expectancy, all DS patients were matched to a random control with same age and sex and the incidence of lung cancer (C34), skin cancer (C44) and breast cancer (C50) were compared between DS patients and control population. All three cancers appear significantly less in DS patients. P-value, CI, df and the chi-squared are noted in the table. CI = confidence interval, df = degree of freedom.

	**P-value (95% CI)**	**df**	**chi-squared**
Lung cancer (C34)	$1.2e^{-2}$ (0.044-0.014)	1	6.25
Skin cancer (C44)	$9.4e^{-3}$ (0.044-0.015)	1	6.73
Breast cancer (C50)	$2.2e^{-4}$ (0.042-0.022)	1	13.63
