# Supplementary material for: Comorbidity landscape of the Danish patient population affected by chromosome abnormalities
Source: Genet Med. 2019 Apr 25;21(11):2485–95. doi: 10.1038/s41436-019-0519-9 (PMC6831512; doi:10.1038/s41436-019-0519-9)
Supplement: Supplementary file 14 — Table S10 [file 41436_2019_519_MOESM14_ESM.pdf]

Comorbidity landscape of the Danish patient population affected by chromosome abnormalities

Isabella Friis Jørgensen, MSc[1, #], Francesco Russo, PhD[1, #], Anders Boeck Jensen, PhD[2], David Westergaard, PhD[1], Mette Lademann, PhD[1], Jessica Xin Hu, PhD[1], Søren Brunak, PhD[1], Kirstine Belling, PhD[1, *]

Supplementary table 10A. Results from comorbidity analysis of non-mosaic DS patients split in 17 groups. The 1050 non-mosaic DS patients were randomly split into groups with 58-62 patients to be able the compare the number of significant comorbidities found, with the 62 mosaic DS patients. As seen in table 1, 16 significant comorbidities were found for the 62 mosaic DS patients, while 17 analyses on the mosaic DS patients showed between 22-33 significant comorbidities, confirming that mosaic DS patients show a more severe phenotype with more significant comorbidities.

Number of non-mosaic DS patients	Number of significant comorbidities
62	26
62	30
62	24
62	27
62	32
62	30
62	28
62	26
62	28
62	33
62	29
62	32
62	27
62	22
62	26
62	28
58	23

Supplementary table 10B. Results from comorbidity analysis of non-mosaic TS patients split in four groups.

The 241 non-mosaic TS patients were randomly split into groups with 58-61 patients to be able the compare the number of significant comorbidities found for the 58 mosaic TS patients. As seen in table 1, one significant comorbidity were found for the 58 mosaic TS patients, while four analyses on the mosaic TS patients showed between 9-16 significant comorbidities, confirming that mosaic TS patients show a more severe phenotype with more significant comorbidities.

Number of non-mosaic TS patients	Number of significant comorbidities
61	9
61	14
61	16
58	16
