# Supplementary material for: Comorbidity landscape of the Danish patient population affected by chromosome abnormalities
Source: Genet Med. 2019 Apr 25;21(11):2485–95. doi: 10.1038/s41436-019-0519-9 (PMC6831512; doi:10.1038/s41436-019-0519-9)
Supplement: Supplementary file 15 — Supplementary text [file 41436_2019_519_MOESM15_ESM.pdf]

Comorbidity landscape of the Danish patient population affected by chromosome abnormalities

Isabella Friis Jørgensen, MSc[1, #], Francesco Russo, PhD[1, #], Anders Boeck Jensen, PhD[2], David Westergaard, PhD[1], Mette Lademann, PhD[1], Jessica Xin Hu, PhD[1], Søren Brunak, PhD[1], Kirstine Belling, PhD[1, *]

Supplementary text 1. Shared direct and inverse comorbidities of aneuploidies

Another common comorbidity between KS and TS patients was infertility: 'Male infertility' (N46) (RR=7.6) for KS and 'Female infertility' (N97) (RR=3.5) for TS. KS and TS patients are known to have fertility problems, which is not the case for DS patients. KS patients are infertile due to a combination of testicular dysfunction and impaired spermatogenesis[1] and most TS patients are infertile due to primary gonadal failure[2] as they typically undergo early menopause due to a fast decline in gonadal function[3]. Female DS patients can be fertile, but the children are often born with DS, congenital malformations or mental retardation. Few DS fathers have been reported and infertility of male DS patients has been described, though the range between sterility and fertility is not clear[4].

The RR for mental retardation was also significantly increased for DS, KS and TS patients. Especially DS patients had an increased risk of 'Mild mental retardation' (F70) (RR=14.7) and 'Unspecified mental retardation' (F79) (RR=111). DS and TS patients also suffered from other mental disorders such as 'Eating disorders' (F50), 'Pervasive developmental disorders' (F84) and 'Other behavioural and emotional disorders with onset usually occurring in childhood and adolescence'

(F98). Other interesting comorbidity overlaps were the high incidence of eye and ear diseases in DS and TS patients. These patients had significant increased RRs for seven eye disorders, including 'Disorders of refraction and accommodation' (H52), 'Visual disturbances' (H53) and 'Visual impairment including blindness' (H54), and for eleven ear diseases, including 'Otitis externa' (H60), 'Nonsuppurative otitis media' (H65), 'Perforation of tympanic membrane' (H72), and 'Conductive and sensorineural hearing loss' (H90).

Early tooth decay was observed for all three chromosome abnormalities, with an increased RR of 'Dental caries' (K02) and 'Dentofacial anomalies' (K07). DS and KS patients were also diagnosed more often with 'Disorders of tooth development and eruption' (K00), 'Diseases of pulp and periapical tissues' (K04) and 'Gingivitis and periodontal diseases' (K05). Significant comorbidities related to decay of bone in general was also observed for KS and TS patients with 'Osteoporosis without pathological fracture' (M81), 'Osteoporosis in diseases classified elsewhere' (M82), and 'Other disorders of bone density and structure' (M85). The RR of 'Osteoporosis in diseases classified elsewhere' (M82) was 34.5 and 146 for TS and KS patients, respectively. Overall, we found that comorbidities common to the three aneuploidies were diabetes, infertility, mental retardation, eye and ear diseases, and tooth and bone decay.

DS is associated with congenital heart disease, but we found DS patients to have a decreased risk of several other heart phenotypes such as: 'Essential (primary) hypertension' (I10) (RR=0.21), 'Angina pectoris' (I20) (RR=0.21), and 'Acute myocardial infarction' (I21) (RR=0.42).

References

1. Lanfranco F, Kamischke A, Zitzmann M, Nieschlag E. Klinefelter's syndrome. *Lancet.* 2004;364(9430):273-283.
2. Abir R, Fisch B, Nahum R, Orvieto R, Nitke S, Ben Rafael Z. Turner's syndrome and fertility: current status and possible putative prospects. *Hum Reprod Update.* 2001;7(6):603-610.

3. Pasquino AM, Passeri F, Pucarelli I, Segni M, Municchi G. Spontaneous pubertal development in Turner's syndrome. Italian Study Group for Turner's Syndrome. *J Clin Endocrinol Metab.* 1997;82(6):1810-1813.
4. Pradhan M, Dalal A, Khan F, Agrawal S. Fertility in men with Down syndrome: a case report. *Fertil Steril.* 2006;86(6):1765 e1761-1763.
